# Oral health and functioning in older adults in Switzerland from 1970 to 2025 - a systematic review and meta-analysis

**DOI:** 10.1007/s00784-026-07105-1

**Published:** 2026-09-03

**Authors:** Roberta Borg-Bartolo, Maurus Jaeggi, Martin Schimmel, Hendrik Meyer-Lueckel

**Affiliations:** 1 Department of Restorative, Pediatric and Preventive Dentistry, School of Dental Medicine, University of Bern, Bern, Switzerland; 2 Department of Reconstructive Dentistry and Gerodontology, School of Dental Medicine, University of Bern, Bern, Switzerland

**Keywords:** Oral health, Epidemiology, Public health, Elderly persons, Functioning, Quality of life, Dental caries

## Abstract

**Objectives:**

This systematic review analysed the oral health status, chewing function, and quality of life among individuals aged ≥ 65 years in Switzerland using studies published from 1970 onwards.

**Materials and methods:**

The electronic databases Embase, MEDLINE *via* PubMed, Swiss data repositories, and manual searches were screened. Risk of bias was assessed using the Joanna Briggs Institute tools. Meta-analyses were performed using the random-effects models with 95% confidence intervals to assess the prevalence of edentulism, periodontal disease, and dental prostheses, as well as the mean number of decayed, missing, and filled teeth and DMFT scores, stratified by living situation and decade.

**Results:**

Across time, edentulism declined markedly (44% in 1980–1990 to 4% in 2020–2025), alongside reductions in the mean number of missing teeth (13.5, 95% CI 12.07–14.93 in 1980–1989 to 5.5 95% CI 3.41–7.59 in 2020–2025). Community dwellers exhibited better oral health status and masticatory performance, while oral hygiene remained moderate to poor across all settings. Very limited evidence was available on oral health-related quality of life, with only three studies reporting patient-reported outcomes.

**Conclusion:**

Overall, oral health among older adults in Switzerland has improved. Across several decades, community-dwelling older adults consistently exhibited better oral health than those living in long-term care facilities (LTF) or hospital settings.

**Supplementary Information:**

The online version contains supplementary material available at https://doi.org/10.1007/s00784-026-07105-1.

## Introduction

Demographic transitions marked by increased life expectancy and declining fertility rates have transformed the age structure of populations worldwide, resulting in a growing proportion of older adults. In Switzerland, approximately 1.8 million individuals are currently aged 65 years or older, with projections estimating an increase to 2.7 million within the next three decades (Swiss Federal Statistical Office, 2025).

Oral diseases are among the most prevalent non-communicable diseases (NCDs) globally [1]. The most common conditions, dental caries and periodontal disease, are chronic and cumulative in nature, and while being highly prevalent, are also preventable. In older adults, these conditions are influenced by a range of interrelated factors, including frailty [2], care-dependency and cognitive decline [3], multi-morbidity, and polypharmacy [4].

Poor oral health can significantly influence systemic health and overall quality of life in older adults. Oral diseases have been associated with nutritional deficiencies and impaired mastication, which can contribute to weight loss and frailty [5]. Furthermore, tooth loss and poor denture function can limit food choices, reducing the intake of fiber, vitamins, and protein, and thereby worsening general health. From a psychosocial perspective, visible tooth loss, halitosis, and ill-fitting dentures can affect self-esteem, social interactions, and mental well-being, leading to social withdrawal and a lower perceived quality of life [6]. Moreover, there is growing evidence linking oral health with systemic conditions such as cardiovascular disease, diabetes mellitus, and respiratory infections [7–9], highlighting the importance of integrating oral health with general health and the need for an interprofessional approach in the management of elderly populations.

Trends in Switzerland have shown that over the past thirty years, there have been improvements in the oral health status of the Swiss population, measured through the decline in the number of missing teeth and an increase in the number of fixed dental restorations as opposed to removable dental restorations [10]. As more older adults retain teeth and have implants, declining dexterity and immune system function make maintenance difficult, increasing the risk of periodontal and peri-implant diseases that can trigger systemic inflammation and worsen chronic conditions [11].

Despite these data, comprehensive national-level evidence on the oral health of older adults in Switzerland remains limited. Most existing studies have been conducted at cantonal or community levels, often focusing on specific subpopulations, or presenting self-reported data only. These fragmented data sources make it difficult to obtain a holistic understanding of the oral health status and needs of older persons in Switzerland.

Given the demographic trajectory and the crucial role of oral health in maintaining general well-being and good quality of life, there is a clear need to synthesize existing evidence to inform policy, research, and clinical practice. To date, no systematic review has comprehensively evaluated the oral health status of older adults in Switzerland. The present review, therefore aims to provide an integrated overview of the current oral health status of elderly persons in Switzerland, and highlight knowledge gaps as well as implications for future oral healthcare provision in an ageing Swiss population.

## Method

### Protocol and registration

This review followed the Preferred Reporting Items for Systematic Reviews and Meta-Analyses (PRISMA) guideline. The review protocol was registered (CRD420251164651) with the International Prospective Register of Systematic Reviews (PROSPERO) system (https://www.crd.york.ac.uk/prospero/).

The CoCoPop framework [12] was applied as follows:

Condition (Co): Oral health status, including:

- Oral hygiene
- Edentulism/tooth loss
- Dental caries
- Periodontal disease
- Chewing function

Oral Health Related Quality of Life (OHRQoL)

Context (Co): time of data collection, place of residence, i.e., community-dwelling, hospital setting, long-term care facilities (LTFs)

Population (Pop): Individuals aged 65 years and older living in Switzerland

### Eligibility criteria

Titles, abstracts and full texts were reviewed according to the following inclusion and exclusion criteria: participants of selected studies had to be aged ≥ 65 years living in Switzerland. No exclusions were made based on where the participants lived, thus the systematic review includes both community-dwellers and nursing home residents. For longitudinal studies, participants had to be aged ≥ 65 years at baseline, as only baseline oral health status data were included (cross-sectional part of the longitudinal study).

Studies reporting the outcomes of participants aged < 65 years and recruited from dental clinics or dental hospitals were excluded from the systematic review. The search was conducted from 1970 onwards and no language restrictions were applied. 1970 was taken as a starting point to include decades where the use of fluoridated toothpaste was widespread. Fluoride-based preventive implementations began in the 1950 s and 1960 s, with widespread use well established in the 1970 s [13].

### Search strategy

The electronic databases Embase, MEDLINE *via* Pubmed, the Swiss repositories of Universities that offer training in dentistry i.e., the University of Basel (University of Basel edoc Repository), University of Bern (BORIS - Portal Bern Open Repository and Information System), University of Geneva (Archive ouverte UNIGE), and the University of Zurich (ZORA - Zurich Open Repository and Archive), and Swisscovery, the national platform comprising scientific information from around 500 libraries in Switzerland, were screened for potentially relevant articles. The search employed both Medical Subject Headings (MeSH) terms and keywords, including: *elderly* and *Switzerland*, the individual variables of interest; *oral hygiene*, *dental caries*, *periodontal disease*, *mastication*, *edentulism*, and *oral health–related quality of life*, and an overall term *oral health* (Supplementary file, Sect. 1). Freehand searches were carried out, including contacting the libraries of the four dental schools in Switzerland to provide any relevant articles related to the systematic review. Journals in the archives of the library of the University of Bern, predating online availability, were searched manually to retrieve potentially relevant articles related to the systematic review. This aided in identifying dissertations of dental students for the conferment of a Doctoral degrees which are generally not available online or readily accessible. The last search was performed in November 2025. Age and publication-type filters were applied during electronic database searches to improve specificity and reduce retrieval of irrelevant records (e.g., case reports, editorials, and studies outside the review scope). Given the comprehensive search strategy, including national and institutional repositories, direct contact with all four Swiss dental schools, and manual archive searches, the likelihood of missing relevant studies was considered minimal.

### Study selection

The retrieved items were imported into Rayyan^®^ [14]. After the removal of duplicate records, two authors (RBB, MJ) independently examined the titles and abstracts, followed by full-text review. Two of the authors (RBB, MJ) independently reviewed the papers, and any disagreements were resolved through discussion. Where this was not possible, two reviewers were consulted (MS, HML). The kappa-statistic was calculated as a measure of interrater agreement between the two raters (RBB, MJ) using StataSE18^®^ (StataCorp LLC USA http://www.stata.com).

### Data extraction and quality assessment

Data extraction and quality assessment were performed independently by the review authors. For each paper, the following data were collected whenever possible: title, author, year of publication, source, country, aim of the study, study design, participant characteristics i.e. age and gender, sample size, and data reported on the oral health status:

- Oral hygiene: any indices used to report oral hygiene status
- Edentulism/tooth loss: tooth loss or edentulism
- Dental caries: dental caries prevalence and severity
- Periodontal disease: any indices used to assess periodontal status
- Chewing function: any objective or subjective method used to evaluate mastication
- OHRQoL: any Patient Reported Outcome Measures (PROMs) used to measure dental Patient Reported Outcomes (dPROs)

Quality assessment was performed using Joanna Briggs Institute (JBI) critical appraisal tools, selected according to study design.

### Statistical analysis

Descriptive analyses were conducted for all included studies. For studies reporting the number of participants and total sample size, meta-analyses of prevalence were performed for edentulism, periodontal disease (defined as pocket depths ≥ 6 mm), and removable and fixed dental prostheses. Analyses were stratified by decade, based on year of data collection (1970–1979, 1980–1989, 1990–1999, 2000–2009, 2010–2019, and 2020–2025) and by setting (community-dwelling populations, hospital settings, and LTFs).

For outcomes reported as the mean number of decayed, missing, and filled teeth and DMFT, studies providing means with standard deviations or medians with interquartile ranges were included in meta-analyses of single means, similarly stratified by decade and setting. Five studies reported means based on a 32-tooth dentition; these were converted to a 28-tooth basis to ensure comparability. The main results are reported on studies originally reporting 28-tooth data. Sensitivity analyses were conducted to assess the impact of the inclusion of the 32-tooth-basis studies.

Random-effects models with 95% confidence intervals were used for the meta-analyses. Heterogeneity was explored using subgroup analyses and meta-regression based on decade, setting, level of data collection (national vs. regional), risk of bias and data-collection methods. Leave-one-out analyses were performed for sensitivity testing. Publication bias was assessed using funnel plots. All statistical analyses were conducted using StataSE 18^®^. Because overall pooled estimates across all studies were not considered clinically meaningful, the overall pooled results are not presented and prediction intervals were not calculated for the overall meta-analyses.

## Results

**Fig. 1:**
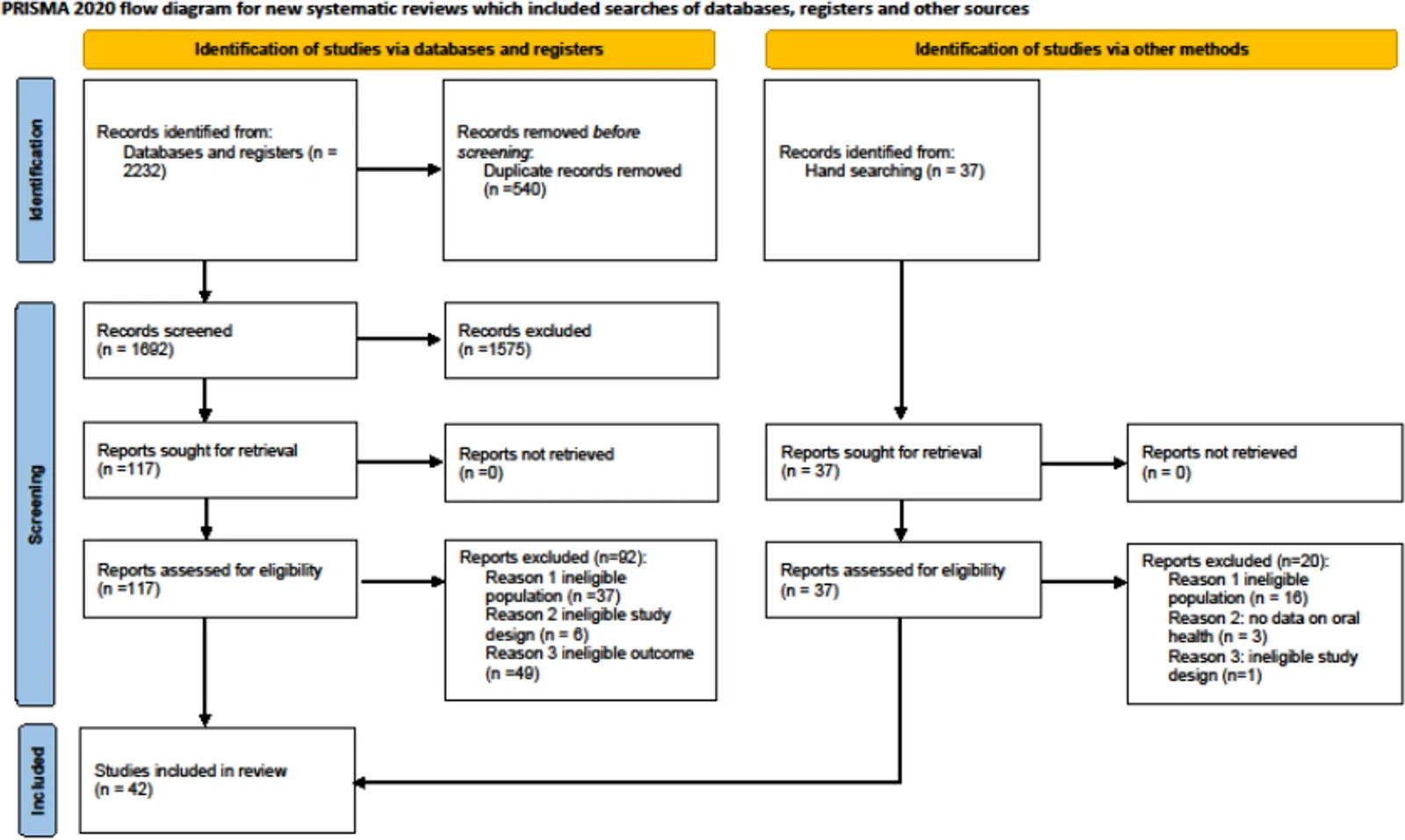
PRISMA flow diagram

After deduplication, the titles and abstracts of papers were reviewed, leaving 154 papers for full-text review. 112 papers were excluded after full-text analysis, resulting in 42 papers for data extraction (Fig. 1).

Where dissertations and published papers reported on the same study, only the published papers were included in the systematic review. In all, 39 studies were included in the systematic review, with 5 papers reporting on the same 2 studies. As they presented different results relevant to the systematic review, all 5 papers were included (Supplementary file, Sect. 3).

The list of references excluded after full-text review and the reason for exclusion are available in the Supplementary file, Sect. 2. Kappa score values for agreement between the two authors who assessed titles and abstracts and full-texts were 0.79 and 0.75, respectively.

### Study characteristics

The number of participants aged 65 years and older in the included studies ranged from 25 [15]−1033 [16]. The majority of the studies were carried out on a cantonal or municipal level and included an intraoral examination. Out of the 42 papers included, 12 were doctoral dissertations. 14 studies were conducted on community-dwellers, eight had participants recruited from a hospital setting, and 17 were conducted on residents of LTFs (Supplementary file, Sect. 3). All data are derived from cross-sectional sources, thus representing temporal trends and differences in living situations.

### Risk of bias

The main concern across most of the studies was whether confounders were identified and whether strategies to address them were reported. While some studies, primarily doctoral dissertations, were purely descriptive and therefore not subject to confounding, other studies did not address potential confounders or did not clearly report whether they had done so (Supplementary file, Sect. 4). The included studies were judged to have an overall low to moderate risk of bias. Meta-regression was performed to test for potential differences between study estimates. Across all meta-analyses, risk of bias was not statistically significant, indicating that study quality did not influence study estimates (Supplementary file, Sect. 5).

### Edentulism

**Fig. 2:**
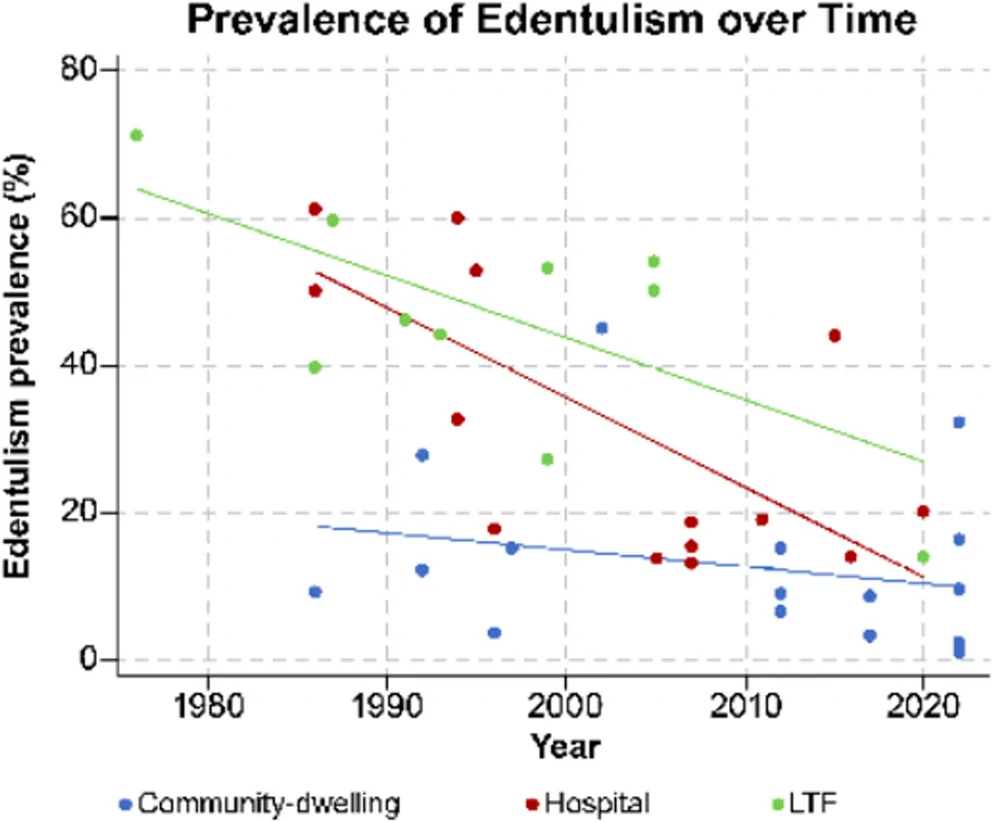
Prevalence of edentulism vs. time

The prevalence of edentulism declined substantially over the 55-year period that was covered by the included studies (Fig. 2).

Meta-analyses stratified by year showed that the prevalence of edentulism decreased from 44% in 1980–1990 (95% CI 27–62%) to 4% in 2020–2025 (95% CI 1–9%). Edentulism prevalence was highest among residents of LTFs (46%, 95% CI 36–56%) and hospital settings (32%, 95% CI 22–43%), and lowest among community-dwelling individuals (7%, 95% CI 3–11%). From approximately the year 2000 onwards, the prevalence of edentulism decreased among older adults irrespective of care setting (Supplementary file, Sect. 5.1).

Meta-regression analyses suggested that the year of data collection accounted for a substantial proportion of between-study variability (57%), with additional contributions from care setting (22%) and level of data collection (8%). Leave-one-out sensitivity analyses indicated that pooled prevalence estimates were robust to the exclusion of individual studies (Supplementary File, Sect. 5.1).

### Dental status

Results were reported for studies calculating mean numbers of decayed (D), missing (M), filled (F), and DMF teeth on a 28-tooth basis. Sensitivity analyses identified statistically significant differences for mean numbers of missing and filled teeth, but no statistically significant differences for mean decayed teeth or mean DMFT, when calculations based on a 32-tooth dentition were included. Forest plots for 28-tooth basis and 32-tooth-basis can be found in the Supplementary file, Sect. 5.2)

### Dental caries

A declining trend in the mean number of decayed teeth over time was observed. Residents of LTFs presented with a higher mean number of decayed teeth than community-dwellers (CD: 0.65, 95% CI 0.29–1.02; LTF: 1.38, 95% CI 0.88–1.88) (Supplementary file, Sect. 5.2.1). Tests to investigate heterogeneity and sensitivity analyses were not performed, as only five studies were included in the analysis.

### Missing teeth

**Fig. 3:**
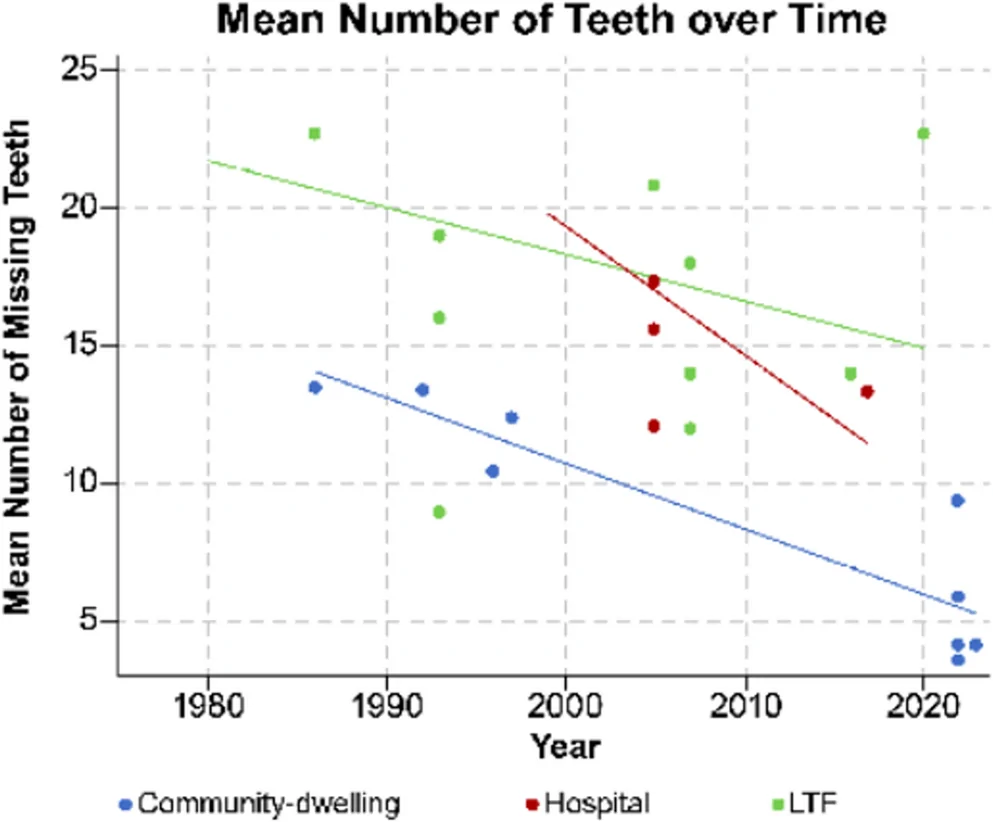
Mean number of missing teeth vs. time

Over time, a decline in the mean number of missing teeth was observed (13.50 (1980–1989), 95% CI 12.70–14.93 to 5.50 (2020–2025), 95% CI 3.41–7.59) (Fig. 3).

Community-dwellers had a lower mean number of missing teeth (8.43, 95% CI 5.77–11.09) than those living in LTFs and hospital settings (16.91, 95% CI 13.54–20.28). Meta-regression analyses indicated that heterogeneity was partly explained by year (27%) and setting (54%). Leave-one-out analyses showed no significant differences in pooled estimates following exclusion of individual studies (Supplementary File, Sect. 5.2.2).

### Filled teeth

Only five studies were included in the meta-analysis. A clear trend could not be observed with regards to changes in filled teeth over time. Community-dwellers presented with a higher mean number of filled teeth than residents of LTFs (CD: 9.81, 95% CI 8.62–11.00; LTF: 3.00, 95% CI 1.74–4.26) (Supplementary file, Sect. 5.2.3). Tests to investigate heterogeneity and sensitivity analyses were not performed, as only five studies were included in the analysis.

### DMFT

In the decades 1980–1989 and 1990–1999, only one study per decade was included in the meta-analysis. A mean DMFT of 25.06 (95% CI 23.62–26.55) was reported in 2010–2019, decreasing to 16.02 (95% CI 11.85–20.19) in the decade 2020–2025. Community-dwellers had a lower mean DMFT (19.33, 95% CI 15.52–23.15) than participants living in LTFs and hospital settings (26.60, 95% CI 25.52–27.72). These results should be interpreted with caution as only 9 studies were included in the subgroup analyses. Meta-regression analyses indicated that year (23%) and setting (36%) partially explained heterogeneity (Supplementary file, Sect. 5.2.4).

### Periodontal disease

**Fig. 4:**
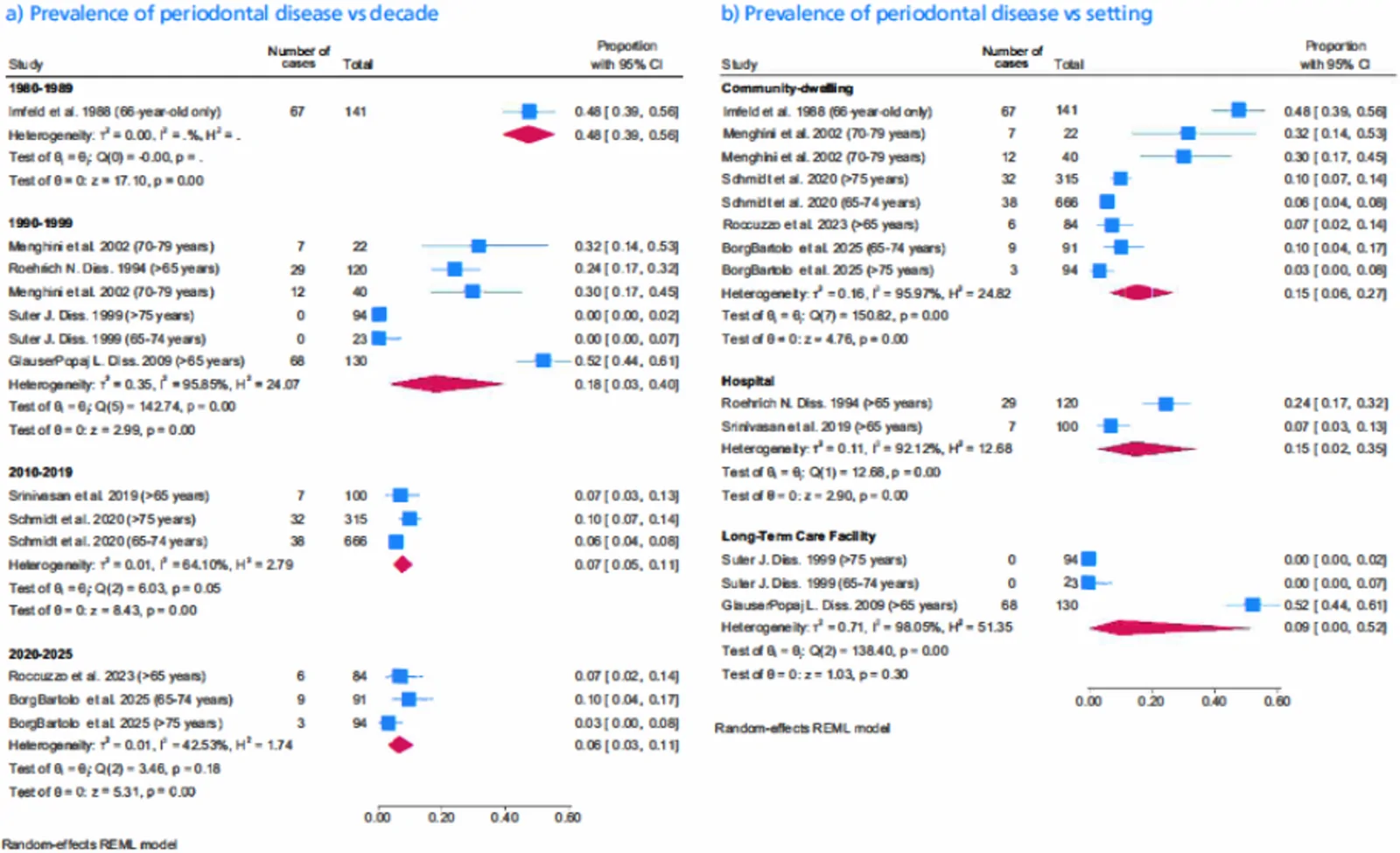
Prevalence of periodontal disease vs. **(a)** decade (**b**) setting

Periodontal assessment varied across the decades, with earlier studies using indices such as Muhlemann and Mazor modified PM-Index [17] and Russell Periodontal index [18] while more recent studies used the Basic Periodontal Examination (BPE), Community Periodontal Index of Treatment Need (CPITN), Periodontal Screening Index (PSI), Periodontal Screening and Recording System (PSR) (Supplementary file, Sect. 3). A declining trend in the prevalence of periodontal disease (pockets of ≥6 mm) was observed across decades, from 18% (95% CI 3–40%) in 1980–1989 to 6% (95% CI 3–11%) in 2020–2025 (Fig. 4a). While a reduction in periodontal disease prevalence was observed in studies of community-dwellers conducted from 2020 onwards, no consistent trend was identified among LTF residents and participants recruited from hospital settings (Fig. 4b). Data from nursing home residents (two studies) showed marked variability, with reported prevalence ranging from 0% (95% CI 0–2%) [19] to 52% (95% CI 44–61%) [20].

Meta-regression analyses indicated that year explained 23% of heterogeneity, whereas setting, method of data collection, and whether data were collected at a national or regional level did not explain heterogeneity (Supplementary File, Sect. 5.3).

### Prosthetic rehabilitation

**Fig. 5:**
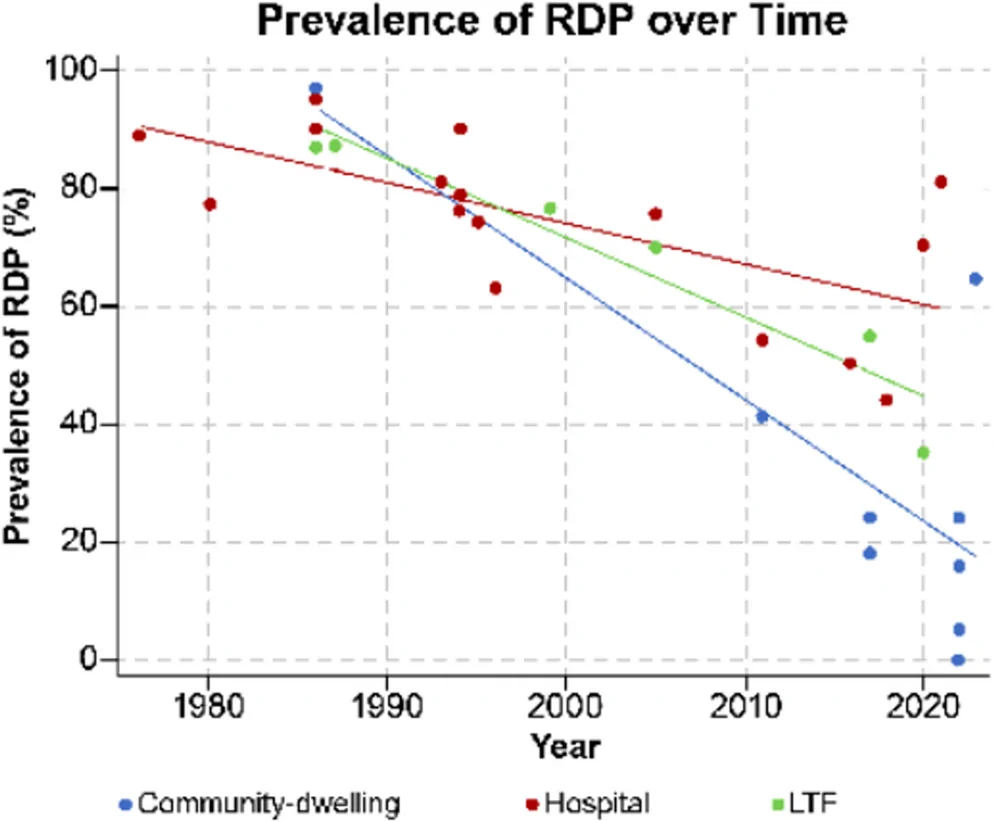
Prevalence of RDP vs. time

The prevalence of removable dental prostheses (RDPs) decreased over time, from 90% (95% CI 83–95%) in 1970–1990 to 20% (95% CI 4–41%) in 2020–2025 (Fig. 5), while the prevalence of fixed dental restorations increased from 13% (95% CI 5–23%) to 81% (95% CI 74–88%) (Supplementary file, Sect. 5.4.2).

Community-dwellers had a lower prevalence of RDP than elderly persons in LTFs and hospital settings (30%, 95% CI 10–55% (CDs) vs. 74%, 95% CI 66–81% (LTFs)) (Fig. 5) and a higher prevalence of fixed dental restorations (62%, 95% CI 36–85% (CDs) vs. 25%, 95% CI 4–54% (LTFs)) (Supplementary file, Sect. 5.4.2).

Meta-regression analyses showed that 61% of heterogeneity was explained by year and 34% by setting in the meta-analysis of RDPs (Supplementary File, Sect. 5.4.1). For fixed dental restorations, heterogeneity was partly explained by year (50%), setting (20%), and method of data collection (13%) (Supplementary file, Sect. 5.4.2).

The prevalence of dental implants was reported in only a few studies (10 [16, 21–25]),. In 1986, Imfeld et al. [21] reported that none of the participants had dental implants. Three studies conducted between 2020 and 2025 reported implant prevalence ranging from 5% [24] to 30% [22], with higher prevalence observed in younger older adults (65–74 years) (10, 18, 19). The Swiss Health Survey 2022 reported a dental implant prevalence of 26.5% among participants aged over 65 years [25].

### Oral hygiene

The most frequently used oral hygiene measures were the Silness and Löe Plaque Index (PI), the Oral Hygiene Index (OHI), and the Approximal Plaque Index (API). Mean PI values fell within the moderate-to-poor range, with mean scores ranging from approximately 1.5 [26] to 2.5 [27] across study populations. Studies using the OHI reported that 75% [28] to 90% [20] of participants were classified as having moderate to poor oral hygiene (Grades 2–3), while studies using the API reported that 33% or more of participants exceeded an API score of 50% or more [22, 23].

### Chewing function

Four studies measured chewing function using the two-color mixing-ability test [29] and assessed the samples either on a nominal scale visually (subjective assessment, SA1 – SA5, where a high SA signifies a good masticatory performance) and opto-electronically by calculating the circular variance of Hue (SD_Hue = 0 - SD_Hue = 1, where a low SD_Hue signifies good masticatory performance) of the Hue-Check Gum© (University of Bern, Switzerland) [23, 30–32]. One study assessed masticatory function by measuring glucose concentration obtained from a gum jelly test to assess masticatory performance using the Gluco sensor GS-II (GC Corporation, Lucerne, Switzerland) [24], while another assessed masticatory ability by self-reported means [33]. Community-dwellers had better chewing function (mean SD_Hue values 0.16 (SD 0.15) [30]) than care-dependent community-dwellers (mean SD_Hue 0.30 (SD 0.20)) ([32]. Elderly patients recruited from a hospital setting presented with a poor chewing function (mean SD_Hue 0.48 (SD 0.25) [31]. Three studies reported the prevalence of poor chewing function;7% (SA 1–2) among community-dwellers [30], 64% in a hospital setting (SA 1–2) [31], and 82% in a rehabilitation and long-term care unit in a hospital setting (glucose concentration > 100 mg/dL) [24].

### Oral health-related quality of life (OHRQoL)

Three studies assessed OHRQoL in community-dwelling older adults. One study used the Oral Health Impact Profile-14 (OHIP-14) [32] and reported a mean score of 8.0 (SD 12.0). Two studies comprising a pilot study [20] and a subsequent main study [32] used the General Oral Health Assessment Index (GOHAI), both reporting a median score of 45 (IQR 30–54; range 23–60) [34]. All OHRQoL scores included in the present systematic review indicate an impact on the quality of life related to oral health.

## Discussion

The present systematic review demonstrates a substantial decline in the prevalence of edentulism in Switzerland over the past five decades, accompanied by reductions in the mean number of missing teeth, and periodontal disease prevalence, alongside an increase in fixed dental restorations. Community-dwelling older adults consistently exhibited better oral health than those recruited from hospital settings or residing in LTFs, a pattern that was also reflected in superior chewing function. In contrast, oral hygiene remained within the moderate to poor range across all settings.

Together, these findings indicate major improvements in tooth retention and restorative dental care over time. School-based dental prevention programmes implemented in many cantons since the mid-20th century [35, 36], combined with fluoride exposure and public oral health campaigns, are likely to have contributed to the favourable effects now visible in today’s older populations. Different generations of older adults would have been exposed throughout their lives to different preventive strategies, patterns of access to dental care, and socioeconomic conditions. Therefore, the findings reflect the broader context of these evolving life-course experiences.

Declining trends in edentulism and tooth loss have similarly been reported internationally [37, 38], a trend also reported by the Swiss Health Survey, which has collected data on the oral health status in persons aged 15 years and older in Switzerland since 1992 [10, 25]. Although Switzerland benefits from nationally collected data on edentulism and tooth loss derived from population-based health surveys using self-reported means, information on dental caries in older adults is considerably more heterogeneous. Caries data are predominantly drawn from clinical examinations conducted within individual cantons or municipalities. While these regional investigations provide valuable and detailed insights into oral health patterns, differences in sampling strategies, age groups, diagnostic criteria, and reporting formats limit direct comparability across regions and over time. For some indicators, namely mean number of decayed and filled teeth, mean DMFT and prevalence of periodontal disease, the available evidence remains limited, with only a few studies included in the meta-analyses, highlighting the need for further high-quality studies before definitive conclusions can be drawn.

Marked differences in oral health status were observed across different living situations. Community-dwelling older adults consistently exhibited better oral health than individuals recruited from hospital environments or residing in LTFs. These disparities extended beyond clinical indicators to functional outcomes, as masticatory performance was also superior among community-dwellers. Such findings align with previous studies suggesting that institutionalized or hospitalized older persons are disproportionately affected by oral disease and tooth loss, potentially owing to higher levels of frailty [39], multimorbidity and polypharmacy [40], functional dependence and cognitive impairments [41], competing medical priorities [42], and possibly barriers to regular dental care [43].

Interestingly, two studies included in the present systematic review have focused on community-dwellers who are care-dependent [32] and elderly persons who have recently been admitted to a LTF [28]. These studies showed that differences exist mostly when care-dependency increases, with the studies both reporting poorer oral health status when compared to participants of similar age groups living in the community with limited or no care-dependency [16, 23].

Despite overall improvements in oral health outcomes, oral hygiene levels among older adults were suboptimal across all settings. This discrepancy between increased tooth retention and persistently suboptimal hygiene highlights an important public health concern. Retaining more natural teeth and complex restorations into advanced age increases the need for meticulous daily oral care, particularly in individuals with reduced manual dexterity, cognitive impairment, or chronic illness, in order to maintain oral health and function. Furthermore, the increasing prevalence of fixed restorations and dental implants, amplifies the need for preventive and supportive periodontal care. These findings underscore the need for targeted preventive programs, caregiver training, and the integration of oral health into routine geriatric and institutional care pathways.

An important finding of this systematic review is the limited evidence on patient-reported oral health outcome measures (PROMs). While the present review demonstrated favourable trends in objective measures, it remains unclear whether these improvements have translated into better perceived oral health or quality of life. While several clinical studies use PROMs, they are notably absent from epidemiological studies. Clinical indicators alone may not fully capture the impact of oral health on daily functioning, psychosocial well-being, or satisfaction with oral health.

This review has several strengths and limitations. Strengths include the absence of language restrictions and an extensive hand search, allowing identification of studies published in multiple languages and those not available online. Limitations arise from substantial heterogeneity between studies, the predominance of cantonal or municipal data, and the limited availability of national clinical data. Oral health outcomes were assessed using diverse methods, including self-report, clinical examinations, and dental records, and some studies reported low response rates, raising concerns about selection bias and representativeness. Such fragmentation particularly complicates assessment of periodontal disease and dental status, which require standardised clinical examinations conducted by trained professionals.

Elderly persons need to be supported in their oral hygiene maintenance, with dental care professionals being aware of the challenges that older people face with their oral health care. Having a good oral health status is essential to avoid oral health issues later on in life, which could be exacerbated by frailty and care dependency associated with greater challenges in oral health care in old age [44, 45]. There are currently non-binding recommendations in Switzerland for older adults who are dependent on care [46]. Strengthening collaboration between dental professionals, nursing staff, and primary care providers, alongside the development of standardized oral health protocols within institutional settings, could help mitigate these disparities and ensure that improvements achieved earlier in life are maintained into advanced age.

Repeated surveys including intra-oral examinations in different age groups following standardized data collection methods on a national level are required as they would allow for the monitoring of oral disease trends and identify areas where improvements are required. Furthermore, further research which is longitudinal in design would allow for causal inference. Greater integration of oral health data with measures of general health and quality of life is also warranted as oral health should not be seen as a separate entity, but an integral part of general health. Future epidemiological studies and national oral health surveillance should therefore routinely incorporate validated PROMs alongside clinical assessments to provide a more comprehensive evaluation of oral health among older adults.

## Conclusion

Overall, the oral health status of the Swiss elderly population has improved over time. Across several decades, community-dwelling older adults consistently exhibited better oral health than those living in LTF or hospital settings. The systematic review highlights the need for standardized research in oral health on a national level.

## Supplementary Information

Below is the link to the electronic supplementary material.

Supplementary Material 1

## Data Availability

Data supporting the findings of this study will be made available by the corresponding author upon reasonable request.

## References

[1] Global Burden of Disease Collaborative Network (2021) Global Burden of Disease Study 2019 (GBD 2019). Institute of Health Metrics and Evaluation (IHME). http://ghdx.healthdata.org/gbd-results-tool

[2] Dibello V, Zupo R, Sardone R, Lozupone M, Castellana F, Dibello A, Daniele A, De Pergola G, Bortone I, Lampignano L, Giannelli G, Panza F (2021) Oral frailty and its determinants in older age: a systematic review. Lancet Healthy Longev 2:e507–e520. 10.1016/S2666-7568(21)00143-4 36098000

[3] Thu Ya M, Hasegawa Y, Sta Maria MT, Hattori H, Kusunoki H, Nagai K, Tamaki K, Hori K, Kishimoto H, Shinmura K (2024) Predicting cognitive function changes from oral health status: a longitudinal cohort study. Sci Rep 14:24153. 10.1038/s41598-024-75169-8 39406928 PMC11480315

[4] Cannon I, Robinson-Barella A, McLellan G, Ramsay SE (2023) From Drugs to Dry Mouth: A Systematic Review Exploring Oral and Psychological Health Conditions Associated with Dry Mouth in Older Adults with Polypharmacy. Drugs Aging 40:307–316. 10.1007/s40266-023-01017-5 36943673

[5] Nitsuwat S, Webster J, Sarkar A, Cade J (2025) The association of oral processing factors and nutrient intake in community-dwelling older adults: a systematic review and meta-analysis. Nutr Rev 83:e762–e777. 10.1093/nutrit/nuae080 38916939 PMC11819486

[6] van de Rijt LJM, Stoop CC, Weijenberg RAF, de Vries R, Feast AR, Sampson EL, Lobbezoo F (2020) The Influence of Oral Health Factors on the Quality of Life in Older People: A Systematic Review. Gerontologist 60:e378–e394. 10.1093/geront/gnz105 31729525

[7] Dorfer C, Benz C, Aida J, Campard G (2017) The relationship of oral health with general health and NCDs: a brief review. Int Dent J 67(Suppl 2):14–18. 10.1111/idj.12360 29023744 PMC9378917

[8] Negrato CA, Tarzia O, Jovanovic L, Chinellato LE (2013) Periodontal disease and diabetes mellitus. J Appl Oral Sci 21:1–12. 10.1590/1678-7757201302106 23559105 PMC3881811

[9] de Souza S, Bansal RK, Galloway J (2017) Managing patients with rheumatoid arthritis. BDJ Team. 10.1038/bdjteam.2017.64

[10] Schneider C, Zemp E, Zitzmann NU (2017) Oral health improvements in Switzerland over 20 years. Eur J Oral Sci 125:55–62. 10.1111/eos.12327 28045197

[11] Muller F, Srinivasan M, Krause KH, Schimmel M (2022) Periodontitis and peri-implantitis in elderly people experiencing institutional and hospital confinement. Periodontol 2000 90:138–145. 10.1111/prd.12454 35916869 PMC9804296

[12] Munn Z, Moola S, Lisy K, Riitano D, Tufanaru C (2015) Methodological guidance for systematic reviews of observational epidemiological studies reporting prevalence and cumulative incidence data. Int J Evid Based Healthc 13:147–153 26317388 10.1097/XEB.0000000000000054

[13] Marthaler T, Marti W, Auer R (1975) Zum Stand der zahnmedizinischen Vorbeugungsbestrebungen in der Schweiz. Sozial- und Präventivmedizin 20:273–277 1229732 10.1007/BF02027409

[14] Ouzzani M, Hammady H, Fedorowicz Z, Elmagarmid A (2016) Rayyan — a web and mobile app for systematic reviews. Syst Rev. 10.1186/s13643-016-0384-4 27919275 PMC5139140

[15] Menghini G, Steiner M, Helfenstein U, Imfeld C, Brodowski D, Hoyer C, Hofmann B, Furrer R, Imfeld T (2002) Zahngesundheit von Erwachsenen im Kanton Zürich. Schweiz Monatsschr Zahnmed 112:708–717 12185725

[16] Schmidt JC, Vogt S, Imboden M, Schaffner E, Grize L, Zemp E, Probst-Hensch N, Zitzmann NU (2020) Dental and periodontal health in a Swiss population-based sample of older adults: a cross-sectional study. Eur J Oral Sci 128:508–517. 10.1111/eos.12738 33073429

[17] Miodragovic M, Schärer P (1980) Mundhygienegewohnheiten, oraler Gesundheitszustand und zahnärztliche Versorgung von Insassen des Kantonalen Krankenheimes Wülflingen. University of Zürich

[18] Stuck A, Flury H, Lang NP (1989) Dental treatment needs in an elderly population referred to a geriatric hospital in Switzerland. Commun Dent Oral Epidemiol 17:267–272 10.1111/j.1600-0528.1989.tb00631.x 2676336

[19] Suter J (1999) Gerostomatologischer Behandlungsbedarf von Altersheim bewohnern in der Schweiz. Friedrich-Schiller-Universität Jena

[20] Glauser-Popaj L (2009) Orale Gesundheit und Mundhygiene von Bewohnern zweier Pflegeheime der Stadt Zürich. Universität Zürich

[21] Imfeld T, Sorg T, Burkhardt P, Engelke W (1988) Zahnärztlicher Befund und Behandlungsnotwendigkeit von 66Jährigen Einwohner der Stadt Zürich. Schweizer Monatsschrift für Zahnmedizin 98:1328–1335 3062774

[22] Roccuzzo A, Borg-Bartolo R, Schimmel M, Tennert C, Manton D, Campus G (2023) Evaluation of the oral health conditions and oral health-related quality of life in a community-dwellers population aged ≥ 45 years in the Canton of Bern: A pilot study. Int J Environ Res Public Health 20:4557. 10.3390/ijerph20054557 36901566 PMC10001686

[23] Borg-Bartolo R, Roccuzzo A, Tennert C, Prasinou M, Jaggi M, Molinero-Mourelle P, Schimmel M, Bornstein MM, Campus G (2025) A Cross-sectional Study Investigating the Oral Health Status of Adult and Elderly Swiss Community-Dwellers. Oral Health Prev Dent 23:67–75. 10.3290/j.ohpd.c_1794 39846968 PMC11801266

[24] Ohta M, Imamura Y, Chebib N, Schulte-Eickhoff RM, Allain S, Genton L, Mojon P, Graf C, Ueda T, Muller F (2022) Oral function and nutritional status in non-acute hospitalised elders. Gerodontology 39:74–82. 10.1111/ger.12612 34913521 PMC9299802

[25] Bundesamt für Statistik (2025) Mund- und Zahngesundheit in der Schweiz, 2002 bis 2022. In: BFS N (ed) Book title. Bundesamt für Statistik (BFS), Neuchâtel

[26] Pazos E (1999) Situation parodontale des personnes âgées en institutions et évaluation d’un programme de prévention. University of Geneva

[27] Mojon PRA, Budtz-Jørgensen E, Baehni PC (1998) Effects of an oral health program on selected clinical parameters and salivary bacteria in a long-term care facility. Eur J Oral Sci 106:827–834. 10.1046/j.0909-8836.1998.eos106401.x 9708685

[28] Brändli-Holzer B (2012) Orale Gesundheit und Mundhygiene von neueingetretenen Bewohnern eines Pflegezentrums der Stadt Zürich. University of Zurich, Faculty of Medicine

[29] Schimmel M, Christou P, Herrmann F, Muller F (2007) A two-colour chewing gum test for masticatory efficiency: development of different assessment methods. J Oral Rehabil 34:671–8. 10.1111/j.1365-2842.2007.01773.x 17716266

[30] Roccuzzo A, Prasinou M, Borg-Bartolo R, Jaeggi MK, Molinero-Mourelle P, Tennert C, Campus G, Schimmel M (2025) Masticatory Performance Within a Representative Swiss Cohort: A Cross-Sectional Study. J Oral Rehabil 52:914–922. 10.1111/joor.13953 39963879

[31] Eggimann AK, Badura L, Zehnder R, Koemeda M, Buser R, Schimmel M (2024) A comparison of oral function in older in- and outpatients: an observational study. Int J Environ Res Public Health. 10.3390/ijerph21080995 39200606 PMC11353424

[32] Angst L, Ferreira Lourenco PD, Srinivasan M (2024) Oral health and nutritional status in care-dependent, community-dwelling older adults in Zurich, Switzerland. Swiss Dent J SSO 134:122–144. 10.61872/sdj-2024-02-09 38739774

[33] Kamdem B, Seematter-Bagnoud L, Botrugno F, Santos-Eggimann B (2017) Relationship between oral health and Fried’s frailty criteria in community-dwelling older person. BMC Geriatr 17. 10.1186/s12877-017-0568-3 PMC5539633 28764647

[34] Borg-Bartolo R, Roccuzzo A, Tennert C, Prasinou M, Jäggi M, Molinero-Mourelle P, Bornstein MM, Campus G (2025) Oral health-related quality of life of community-dwellers in the canton of Bern, Switzerland. Acta Odontol Scand 84:26–36. 10.2340/aos.v84.42707

[35] Steiner M, Menghini G, Marthaler T, Imfeld T (2010) Changes in dental caries in Zurich school-children over a period of 45 years. Schweiz Monatsschr Zahnmed 120:1084–1094 21243546

[36] Magri F, Richle B (2013) Schulzahnpflege Vademecum für den Einsatz von SZPI. Accessed

[37] Kassebaum NJ, Bernabe E, Dahiya M, Bhandari B, Murray CJ, Marcenes W (2014) Global Burden of Severe Tooth Loss: A Systematic Review and Meta-analysis. J Dent Res 93:20S–28S. 10.1177/0022034514537828 24947899 PMC4293725

[38] Müller F, Naharro M, Carlsson GE (2007) What are the prevalence and incidence of tooth loss in the adult and elderly population in Europe? Clin Oral Implants Res 18(Suppl 3):2–14. 10.1111/j.1600-0501.2007.01459.x 17594365

[39] Niesten D, Witter DJ, Bronkhorst EM, Creugers NHJ (2017) Oral health care behavior and frailty-related factors in a care-dependent older population. J Dent 61:39–47. 10.1016/j.jdent.2017.04.002 28380347

[40] Nakamura JKK, Ueda Y, Nishio E, Shibatsuji T, Uchihashi Y, Adachi R, Ono R (2021) Impact of polypharmacy on oral health status in elderly patients admitted to the recovery and rehabilitation ward. Geriatr Gerontol Int 21:66–70 33280240 10.1111/ggi.14104

[41] Jockusch J, Riese F, Theill N, Sobotta BAJ, Nitschke I (2021) Aspects of oral health and dementia among Swiss nursing home residents. Z Gerontol Geriatr 54:500–506. 10.1007/s00391-020-01739-w 32488304

[42] Baumgartner W, Schimmel M, Müller F (2015) Oral health and dental care of elderly adults dependent on care. SWISS Dent J SSO 125:417–426 10.61872/sdj-2015-04-01 26169068

[43] Beaven A, Marshman Z (2024) Barriers and facilitators to accessing oral healthcare for older people in the UK: a scoping review. Br Dent J. 10.1038/s41415-024-7740-x 39152268

[44] Fisher M, Ghezzi E (2013) Preparing Patients for Future Oral Healthcare Decline: What Dentists Can Do Today. Compendium of Continuing Education in Dentistry, p 34 23556323

[45] Pretty IA, Ellwood RP, Lo EC, MacEntee MI, Müller F, Rooney E, Murray Thomson W, Van der Putten GJ, Ghezzi EM, Walls A, Wolff MS (2014) The Seattle Care Pathway for securing oral health in older patients. Gerodontology 31:77–87. 10.1111/ger.12098 24446984

[46] Jiang CM, Chu CH, Duangthip D, Ettinger RL, Hugo FN, Kettratad-Pruksapong M, Liu J, Marchini L, McKenna G, Ono T, Rong W, Schimmel M, Shah N, Slack-Smith L, Yang SX, Lo ECM (2021) Global perspectives of oral health policies and oral healthcare schemes for older adult populations. Front Oral Health 2:703526. 10.3389/froh.2021.703526 35048040 PMC8757822

