# Supplementary material for: Oral health and functioning in older adults in Switzerland from 1970 to 2025 - a systematic review and meta-analysis
Source: Clin Oral Investig. 2026 Sep 3;30(9):428. doi: 10.1007/s00784-026-07105-1 (PMC13538167; doi:10.1007/s00784-026-07105-1)
Supplement: Supplementary file 1 [file 784_2026_7105_MOESM1_ESM.docx]

Oral health and functioning

in older adults in Switzerland

from 1970-2025

- a systematic review and meta-analysis

Roberta Borg-Bartolo^1^, Maurus Jäggi^1^, Martin Schimmel^2^, Hendrik Meyer-Lückel^1^

^1^Department of Restorative, Pediatric and Preventive Dentistry, School of Dental Medicine, University of Bern, Bern, Switzerland

^2^Department of Reconstructive Dentistry and Gerodontology, School of Dental Medicine, University of Bern, Bern, Switzerland

Corresponding author: Roberta Borg-Bartolo: roberta.borg-bartolo@unibe.ch

Contents

[Section 1: Search strategy for each electronic database	3](#_Toc222153519)

[Section 2: Studies excluded after full-text review	5](#_Toc222153520)

[Section 3: Characteristics of Included Studies	14](#_Toc222153521)

[Section 4. Quality Assessment	19](#_Toc222153522)

[Section 5. Data synthesis	21](#_Toc222153523)

# Section 1: Search strategy for each electronic database

# *the search strategy is shown for the overall term ‘oral health’. Individual searches were performed for each variable of interest

| **Pubmed** | **(("oral health"[MeSH Terms] OR ("oral"[All Fields] AND "health"[All Fields]) OR "oral health"[All Fields]) AND ("aged"[MeSH Terms] OR "aged"[All Fields] OR "elderly"[All Fields] OR "elderlies"[All Fields] OR "elderly s"[All Fields] OR "elderlys"[All Fields]) AND ("switzerland"[MeSH Terms] OR "switzerland"[All Fields] OR "switzerland s"[All Fields])) AND ((aged[Filter] OR 80andover[Filter]) AND (1970:2025[pdat]))** Filters: **Books and Documents, Classical Article, Clinical Study, Comparative Study, Evaluation Study, Interview, Multicenter Study, Observational Study, Randomized Controlled Trial, Validation Study, 80 and over: 80+ years, Aged: 65+ years**  (("oral health"[MeSH Terms] OR ("oral"[All Fields] AND "health"[All Fields]) OR "oral health"[All Fields]) AND ("aged"[MeSH Terms] OR "aged"[All Fields] OR "elderly"[All Fields] OR "elderlies"[All Fields] OR "elderly s"[All Fields] OR "elderlys"[All Fields]) AND ("switzerland"[MeSH Terms] OR "switzerland"[All Fields] OR "switzerland s"[All Fields]) AND (("aged"[MeSH Terms] OR "aged, 80 and over"[MeSH Terms]) AND 1970/01/01:2025/12/31[Date - Publication])) AND ((booksdocs[Filter] OR classicalarticle[Filter] OR clinicalstudy[Filter] OR comparativestudy[Filter] OR evaluationstudy[Filter] OR interview[Filter] OR multicenterstudy[Filter] OR observationalstudy[Filter] OR randomizedcontrolledtrial[Filter] OR validationstudy[Filter]) AND (80andover[Filter] OR aged[Filter])) |
| --- | --- |
| **Embase** | ('oral health'/exp OR 'oral health' OR (oral AND ('health'/exp OR health))) AND 'aged':ti,ab,kw AND 'switzerland':ti,ab,kw AND (1976:py OR 1978:py OR 1980:py OR 1988:py OR 1989:py OR 1990:py OR 1991:py OR 1992:py OR 1994:py OR 1996:py OR 1997:py OR 1998:py OR 1999:py OR 2000:py OR 2001:py OR 2002:py OR 2003:py OR 2004:py OR 2005:py OR 2006:py OR 2007:py OR 2008:py OR 2009:py OR 2010:py OR 2011:py OR 2012:py OR 2013:py OR 2014:py OR 2015:py OR 2016:py OR 2017:py OR 2018:py OR 2019:py OR 2020:py OR 2021:py OR 2022:py OR 2023:py OR 2024:py OR 2025:py) AND ('clinical article'/de OR 'clinical study'/de OR 'cohort analysis'/de OR 'comparative study'/de OR 'controlled clinical trial'/de OR 'controlled study'/de OR 'cross sectional study'/de OR 'evidence based practice'/de OR 'intervention study'/de OR 'interview'/de OR 'longitudinal study'/de OR 'major clinical study'/de OR 'multicenter study'/de OR 'observational study'/de OR 'pilot study'/de OR 'prospective study'/de OR 'qualitative research'/de OR 'questionnaire'/de OR 'randomized controlled trial'/de OR 'retrospective study'/de OR 'validation study'/de) AND ('aged':ag OR 'very elderly':ag) |

# Section 2: Studies excluded after full-text review

| **Author** | **Title** | **Reason for exclusion** |
| --- | --- | --- |
| Abou-Ayash et al 2022 | Converting Bilateral Free-End Removable Partial Dentures to Implant-Assisted Removable Partial Dentures Using 6 mm Short Implants: Patient-Reported Outcomes of a Prospective Clinical Study | no data on oral health status |
| Alchalabi et al 2024 | Removable dental prostheses and difficulties with chewing among frail individuals: Results from the Swiss SAPALDIA cohort | population does not match inclusion criteria |
| Aquilanti et al 2020 | Impact of Elderly Masticatory Performance on Nutritional Status: An Observational Study | population does not match inclusion criteria |
| Aslund et al 2008 | Measuring oral health-related quality-of-life using OHQoL-GE in periodontal patients presenting at the University of Berne | population does not match inclusion criteria |
| [Bakker et al 2018](https://pubmed.ncbi.nlm.nih.gov/?size=50&term=Bakker+MH&cauthor_id=30545100) | Are edentulousness, oral health problems and poor health-related quality of life associated with malnutrition in community-dwelling elderly (aged 75 years and over)? A cross-sectional study | population does not match inclusion criteria |
| Baumgartner et al 2015 | Oral health and dental care of elderly adults dependent on care | no data on oral health status |
| Bleiel et al 2022 | Impact of care level, setting and accommodation costs on a newly developed oral care nursing plan format for elderly patients with care needs – Results from a cross-sectional study | population does not match inclusion criteria |
| Bolliger et al 2002 | Influence of long-term smoking reduction on health risk markers and quality of life | no data on oral health status |
| Bornstein et al 2009 | Prevalence of halitosis in the population of the city of Bern, Switzerland: a study comparing self-reported and clinical data | population does not match inclusion criteria |
| Bourgeois et al 1998 | Prevalence of caries and edentulousness among 64-74-years-old in Europe | secondary data |
| [Bousiou et al 2022](https://pubmed.ncbi.nlm.nih.gov/?size=50&term=Bousiou+A&cauthor_id=34850264) | Sociomedical and oral factors affecting masticatory performance in an older population | population does not match inclusion criteria |
| Brunner et al 1987 | Die Zahnmedizinische Betreuung in den Alters- und Pflegeheimen des Kantons Zürich | population does not match inclusion criteria |
| Brunner et al 1989 | Late results with hybrid dentures in low-income patients. An investigation at the Cantonal Public Dental Clinic of Zurich | population does not match inclusion criteria |
| Busin 1990 | Mundhygienegewohnheiten, oraler Gesundheitszustand und zahnärztliche Versorgung von über 50jährigen minderbemittelten Erwachsenen, die sich in der Kantonalen Volkszahnklinik Zürich zur Kontrolle und Behandlung meldeten | population does not match inclusion criteria |
| Büttel et al 2012 | Immediate loading of two unsplinted mandibular implants in edentulous patients with an implant-retained overdenture: an observational study over two years | no data on oral health status |
| Choi et al 2021 | Factors Influencing Oral Health-Related Quality of Life in Older Adults in Rural Areas: Oral Dryness and Oral Health Knowledge and Behavior | population does not match inclusion criteria |
| Czwikla et al 2021 | Effectiveness of a Dental Intervention to Improve Oral Health among Home Care Recipients: A Randomized Controlled Trial | population does not match inclusion criteria |
| de Sá et al 2025 | The Effect of Implant Distribution on Functional and Patient-Reported Outcomes of Mandibular Overdentures Retained by Four Mini Implants | no data on oral health status |
| Deppe et al 2015 | Erosive esophageal reflux vs. non erosive esophageal reflux: oral findings in 71 patients | no data on oral health status |
| Donker et al 2024 | Immediate implant placement with immediate or delayed provisionalization in the maxillary aesthetic zone: A 10-year randomized trial | no data on oral health status |
| Dormenval et al 1998 | Associations between malnutrition, poor general health and oral dryness in hospitalized elderly patients | no data on oral health status |
| Egloff 1988 | Die zahnmedizinische Betreuung in den Alters- und Pflegeheimen sowie den medizinisch-geriatrischen Kliniken der Kantone Basel-Stadt und Basel-Land | no data on oral health status |
| Elsig et al 2015 | Tooth loss, chewing efficiency and cognitive impairment in geriatric patients | population does not match inclusion criteria |
| Engelberger et al 1970 | Parodontal Knochenschwund und Kariesbefall bei Erwachsene | population does not match inclusion criteria |
| Enkling et al 2020 | Clinical Outcomes and Bone-Level Alterations around One-Piece Mini Dental Implants Retaining Mandibular Overdentures: 5-Year Follow-up of a Prospective Cohort Study. | no data on oral health status |
| Enkling et al 2021 | Short Strategic Implants for Mandibular Removable Partial Dentures: One-Year Results from a Pilot Randomized Crossover Abutment Type Study | no data on oral health status |
| Enkling et al 2022 | Influence of the Loading Protocol and Platform Switching in Two-Implant Bar-Retained Overdentures: 3-Year Results from a Randomized Controlled Equivalence Clinical Trial. | no data on oral health status |
| Feil 1988 | Zahnärztlicher Befund bei Pensionären privater Altersheime der Stadt St. Gallen | population does not match inclusion criteria |
| Feldmann 1989 | Orale Zustand bei Erwachsenen in der Schweiz. Eine Erhebung in 47 zufällig ausgewählten Praxen. | population does not match inclusion criteria |
| Franscini 1984 | Die zahnärztliche Betreuung in den Alters- und Pflegeheimen des Kantons Zürich | no data on oral health status |
| Frei et al 2012 | Assessment of smoking behaviour in the dental setting. A study comparing self-reported questionnaire data and exhaled carbon monoxide levels | no data on oral health status |
| Gagesch et al 2023 | Feasibility of a 12-Month Follow-Up in Swiss Older Adults after Post-Acute Care in Nursing Homes—A Pilot Study | population does not match inclusion criteria |
| Gisler et al 2012 | A cross-sectional analysis of the prevalence of dental anxiety and its relation to the oral health-related quality of life in patients with dental treatment needs at a university clinic in Switzerland | population does not match inclusion criteria |
| Glenz et al 2015 | The elderly patient: no reason to worry!? The need for health screening in elderly and very old patients in daily practice | narrative review |
| Goetz et al 2001 | Test-retest reliability of a German language multidimensional assessment instrument in elderly probands | population does not match inclusion criteria |
| Hägler et al 1970 | Rückgang des Karieszuwachses durch systematische Vorbeugung beim Erwachsenen | population does not match inclusion criteria |
| Hajek et al 2021 | The association between oral health-related quality of life, loneliness, perceived and objective social isolation: results of a nationally representative survey | population does not match inclusion criteria |
| Hajek et al 2022 | Does Oral Health-Related Quality of Life Differ by Income Group? Findings from a Nationally Representative Survey | population does not match inclusion criteria |
| Hartmann et al 2020 | A Parallel 3-Group Randomised Clinical Trial Comparing Different Implant Treatment Options for the Edentulous Mandible: 1-Year effects on Dental Patient-Reported Outcomes and Chewing Function | no data on oral health status |
| Hassel et al 2011 | Oral health-related quality of life is linked with subjective well-being and depression in early old age | population does not match inclusion criteria |
| Heimann 1993 | Untersuchungen zur Gebiss- und Mundgesundheit bei älteren Personen in Alten- und Pflegeheimen : Teil I: Karies- und Parodontalbefunde | population does not match inclusion criteria |
| Holtfreter et al 2012 | A comparison of periodontal status in the two regional population-based studies of SHIP and INVEST | population does not match inclusion criteria |
| Huang et al 2025 | Modelling analysis of dietary behaviors and oral health status to assess the impact on the mental well-being of elderly individuals: a cross-sectional survey study | population does not match inclusion criteria |
| Ikebuchi et al 2021 | Relationship between Masticatory Function and Bone Mineral Density in Community-Dwelling Elderly: A Cross-Sectional Study | population does not match inclusion criteria |
| Imamura et al 2023 | Masticatory performance in oral function assessment: alternative methods. | population does not match inclusion criteria |
| Imfeld 1991 | Zahnbestand in Relation zum Alter: Erhebungen in der Schweiz 1980-1990 | secondary data |
| Iorio-Siciliano et al 2023 | Resolution of Peri-Implant Mucositis at Tissue-and Bone-Level Implants: A 6-Month Prospective Controlled Clinical Trial | no data on oral health status |
| Jabir et al 2021 | Evaluating the effectiveness of fluoride varnish in preventing caries amongst Long‐Term Care Facility Residents | population does not match inclusion criteria |
| Jetzer et al 1980 | Zahnverlust und prothetische Versorgung in Abhängigkeit Alter bei der Schweizer Bevölkerung | population does not match inclusion criteria |
| Jockusch et al 2020 | Aspects of oral health and dementia among Swiss nursing home residents | population does not match inclusion criteria |
| Jockusch et al 2020 | Retrospective Analysis of Oral Health Status of Swiss Nursing Home Residents | population does not match inclusion criteria |
| Jockusch et al 2021 | Chewing function and related parameters as a function of the degree of dementia: Is there a link between the brain and the mouth? | population does not match inclusion criteria |
| Jockusch et al 2021 | Influence of cognitive impairment and dementia on oral health and the utilization of dental services : Findings of the Oral Health, Bite force and Dementia Study (OrBiD) | population does not match inclusion criteria |
| Jockusch et al 2022 | The Effect of a Masticatory Muscle Training Program on Chewing Efficiency and Bite Force in People with Dementia | population does not match inclusion criteria |
| Jockusch et al 2022 | Impact of an Oral Hygiene Intervention in People with and without Dementia on Oral Health Parameters-Results from the Oral Health, Bite Force, and Dementia (OrBiD) Pilot Study | population does not match inclusion criteria |
| Jünger et al 2020 | Anti-inflammatory potential of an essential oil-containing mouthwash in elderly subjects enrolled in supportive periodontal therapy: a 6-week randomised controlled clinical trial | no data on oral health status |
| Katsoulis et al 2012 | Oral and general health status in patients treated in a dental consultation clinic of a geriatric ward in Bern Switzerland | population does not match inclusion criteria |
| [Kim et al 2021](https://pubmed.ncbi.nlm.nih.gov/?size=50&term=Kim+HE&cauthor_id=33919497) | Influential factors of masticatory performance in older adults: A cross-sectional study | population does not match inclusion criteria |
| [Kusunoki et al 2024](https://pubmed.ncbi.nlm.nih.gov/?size=50&term=Kusunoki+H&cauthor_id=39796450) | Oral Frailty and Its Relationship with Physical Frailty in Older Adults: A Longitudinal Study Using the Oral Frailty Five-Item Checklist | population does not match inclusion criteria |
| Laugisch et al 2021 | Oral and Periodontal Health in Patients with Alzheimer's Disease and Other Forms of Dementia - A Cross-sectional Pilot Study. | population does not match inclusion criteria |
| [Lee et al 2023](https://pubmed.ncbi.nlm.nih.gov/?size=50&term=Lee+NJ&cauthor_id=36367631) | Assessment of subjective and objective masticatory function among elderly individuals with mild cognitive impairment | population does not match inclusion criteria |
| Leles et al 2024 | Changes in masticatory performance and bite force after treatment with mandibular overdentures retained by four titanium-zirconium mini implants: One-year randomised clinical trial | no data on oral health status |
| Lourenço et al 2024 | Quality of nutritional uptake of community-dwelling, care-dependent older adults in Zurich | no data on oral health status |
| Mancini et al 2024 | Impact of keratinized mucosa on implant-health related parameters: A 10-year prospective re-analysis study | no data on oral health status |
| Maniewicz et al 2019 | Effect of Implant-Supported Mandibular Overdentures versus Reline on Masticatory Performance and Salivary Flow Rates in Very Old Adults-A Randomized Clinical Trial | no data on oral health status |
| Manski et al 2015 | Disparity in dental coverage among older adult populations: a comparative analysis across selected European countries and the USA | no data on oral health status |
| Marenbach, 2005 | Gerostomatologische Untersuchung zur Mundgesundheit und Lebensqualität hospitalisierter Menschen | population does not match inclusion criteria |
| Marthaler 1978 | Ist Zahnlosigkeit im Zurückgehen begriffen? | population does not match inclusion criteria |
| Meyer et al 2017 | Experimental Mucositis and Experimental Gingivitis in Persons Aged 70 or over. Clinical and Biological Responses | no data on oral health status |
| Miolin et al 2007 | Clinical effectiveness of two different toothbrushes in the elderly | no data on oral health status |
| Mojon et al 1994 | Estimates of time and propensity for dental treatment among institutionalised elders | population does not match inclusion criteria |
| Müller et al 2015 | Small-Diameter Titanium Grade IV and Titanium-Zirconium Implants in Edentulous Mandibles: Five-Year Results from a Double-Blind Randomized Controlled Trial. | no data on oral health status |
| Müller et al 2022 | Periodontitis and peri-implantitis in elderly people experiencing institutional and hospital confinement | narrative review |
| Netzle 1989 | Zahnbefund bei Hochbetagten Heimpensionären | population does not match inclusion criteria |
| Nika, 2011 | Untersuchungen zum Mundgesundheitszustand älterer Patienten und dereen mundgesundheitsbezogene Lebensqualität | population does not match inclusion criteria |
| Nitschke et al 2010 | Dental care of frail older people and those caring for them | population does not match inclusion criteria |
| Nitschke et al 2021 | Health-Related Social and Ethical Considerations towards the Utilization of Dental Medical Services by Seniors: Influencing and Protective Factors, Vulnerability, Resilience and Sense of Coherence | narrative review |
| Nitschke et al 2024 | Influence of visual acuity, manual dexterity and handgrip strength on oral and denture hygiene ability of non-frail older people: Development of the Gerostomatological Assessment Battery | population does not match inclusion criteria |
| [Noetzel et al 2021](https://pubmed.ncbi.nlm.nih.gov/?size=50&term=Noetzel+N&cauthor_id=33206351) | The impact of oral health on prognosis of older multimorbid inpatients: the 6-month follow up MPI oral health study (MPIOH) | population does not match inclusion criteria |
| Ramseier et al 2021 | Effect of Age on Bleeding on Probing (BOP) as an Indicator of Periodontal Inflammation in Patients Enrolled in Supportive Periodontal Therapy | no data on oral health status |
| Reissmann et al 2018 | Long-Term Changes in Oral Health-Related Quality of Life over a Period of 5 Years in Patients Treated with Narrow Diameter Implants: A Prospective Clinical Study. | no data on oral health status |
| Reissmann et al 2023 | IMPACT OF LOADING PROTOCOL OF 2-IMPLANT BAR-RETAINED MANDIBULAR OVERDENTURES ON ORAL HEALTH-RELATED QUALITY OF LIFE: A RANDOMIZED CONTROLLED TRIAL | no data on oral health status |
| Renvert et al 2024 | The efficacy of reconstructive therapy in the surgical management of peri-implantitis: A 3-year follow-up of a randomized clinical trial | no data on oral health status |
| Resende et al 2021 | Single versus Two-Implant Mandibular Overdentures Using Early-Loaded Titanium-Zirconium Implants with Hydrophilic Surface and Ball Attachments: 1-Year Randomized Clinical Trial. | no data on oral health status |
| Rodriguez et al 2017 | Lower educational level is a risk factor for tooth loss - Analysis of a Swiss Population (KREBS Project) | population does not match inclusion criteria |
| Rutar et al 2001 | Retrospective assessment of clinical and microbiological factors affecting periimplant tissue conditions | no data on oral health status |
| Sato et al 2025 | Association between self-reported difficulty in chewing or swallowing and frailty in older adults: A retrospective cohort study | population does not match inclusion criteria |
| Schenk et al 2024 | One-Piece Mini Dental Implant-Retained Mandibular Overdentures: 10-Year Clinical and Radiological Outcomes of a Non-Comparative Longitudinal Observational Study | no data on oral health status |
| Schimmel et al 2008 | Utilisation of dental services in a university hospital palliative and long-term care unit in Geneva | no data on oral health status |
| Schürch et al 2015 | Trends of periodontal conditions in two different randomly selected Swiss (Bernese) cohorts 25 years apart | population does not match inclusion criteria |
| [Shiota et al 2023](https://pubmed.ncbi.nlm.nih.gov/?size=50&term=Shiota+C&cauthor_id=37892444) | Oral Hypofunction and Risk of Weight Change among Independent Older Adults | population does not match inclusion criteria |
| Shirobe et al 2022 | Effect of an Oral Frailty Measures Program on Community-Dwelling Elderly People: A Cluster-Randomized Controlled Trial | population does not match inclusion criteria |
| Siegmund 2001 | Untersuchungen zu Wechselwirkungen zwischen der Mundgesundheit und gastrointestinalen Krankheiten älterer Menschen während der Durchführung von Rehabilitationsmassnahmen | population does not match inclusion criteria |
| Srinivasan et al 2014 | High-fluoride toothpaste: a multicenter randomized controlled trial in adults | no data on oral health status |
| Srinivasan et al 2020 | Does a Sage-Based Mouthwash Improve OHRQoL and Xerostomia in Elders? | no data on oral health status |
| Srinivasan et al 2020 | Mandibular two-implant overdentures with CAD-CAM milled bars with distal extensions or retentive anchors: A randomized controlled trial | no data on oral health status |
| Srinivasan et al 2025 | Comprehensive Pilot Analysis of Oral Hypofunction Among Swiss Adults | population does not match inclusion criteria |
| Stadelmann et al 2012 | Dental visits oral hygiene behaviour and orthodontic treatment in Switzerland | no data on oral health status |
| Stalder et al 2021 | Biological and Technical Complications in Root Cap-Retained Overdentures after 3-15 yars in Situ: A Retrospective Clinical Study. | no data on oral health status |
| Stillhart et al 2025 | Thermographic images for screening oral health problems in older adults: A pilot study | No data for oral health status |
| Stock et al 2016 | A comparison of tooth retention and replacement across 15 countries in the over-50s | secondary data |
| Suhm et al 2008 | Management of fragility fractures in Switzerland: results of a nationwide survey | no data on oral health status |
| Swoboda et al 2006 | Predictors of Oral Health Quality of Life in Older Adults | population does not match inclusion criteria |
| [Tani et al 2023](https://pubmed.ncbi.nlm.nih.gov/?size=50&term=Tani+A&cauthor_id=37049448) | The Impact of Nutrition and Oral Function Exercise on among Community-Dwelling Older People | population does not match inclusion criteria |
| Tennert et al 2020 | An Oral Health Optimized Diet Reduces the Load of Potential Cariogenic and Periodontal Bacterial Species in the Supragingival Oral Plaque: A Randomized Controlled Pilot Study | no data on oral health status |
| Tristiu et al 2018 | Association of Oral-Health Related Quality of Life and General Health Assessment in Patients with Rheumatoid Arthritis | no data on oral health status |
| von der Mühll 1997 | Situation sociale et status bucco-dentaire d'un groupe de patients hospitalisés à l'Hôpital de gériatrie de Genève évalués à l'aide d'un questionnaire | no data on oral health status |
| Wallace et al 2018 | Impact of prosthodontic rehabilitation on the masticatory performance of partially dentate older patients: Can it predict nutritional state? Results from a RCT | no data on oral health status |
| Witte, 1986 | Klinische Studie zur Evaluation von Zusammenhängen zwischen soziodemographischen Parametern (Alter), psychosozialen Einflüssen (psychischer Gesundheitszustand) sowie der Mobilität der Patienten (physischer Gesundheitszustand) zum Mundhygienestatus bei Patienten über 70 Jahren | population does not match inclusion criteria |
| [Ye et al 2024](https://pubmed.ncbi.nlm.nih.gov/?size=50&term=Ye+N&cauthor_id=39484361) | The association between oral health and mild cognitive impairment in community-dwelling older adults | population does not match inclusion criteria |
| Zembic et al 2014 | Patient-reported outcomes of maxillary implant-supported overdentures compared with conventional dentures | no data on oral health status |
| Zembic et al 2019 | Patient-Reported Outcomes of Maxillary Edentulous Patients Wearing Overdentures Retained by Two Implants from Insertion to 4 Years | no data on oral health status |
| Ziebolz et al 2017 | Oral Health and nutritional status in nursing home residents-results of an explorative cross-sectional pilot study | population does not match inclusion criteria |

# Section 3: Characteristics of Included Studies

| **Study** | **Data collection** | **Sample size (≥65 years)** | **Data collection** | **Dental caries** | **Periodontal disease** | **Edentulism** | **Oral hygiene** | **Dental prostheses** | | | **Mastication** | **OHRQoL** |
| --- | --- | --- | --- | --- | --- | --- | --- | --- | --- | --- | --- | --- |
|  |  |  |  |  |  |  |  | **removable** | **fixed** | **implants** |  |  |
| **Community Dwellers** | | | | | | | | | | | | |
| Angst et al. 2024 | Regional | 275 | oral examination | DMFT | CPITN | + | PI/GI |  |  |  | SA, SD_Hue | OHIP-14 |
| Borg Bartolo et al. 2025* | Regional | 197 | oral examination | DMFT | PSI | + | API, mPBI | + | + | + | - | - |
| Borg-Bartolo et al. 2025* | Regional | 197 | oral examination | ICDAS, DMFT | PSI | - | API, mPBI |  |  |  | SA, SD_Hue | GOHAI |
| Bundesamt für Statistik 2025 | National | NA | self-reported | - | - | - | - | + | - | + | - | - |
| Eggimann et al. 2024 | Regional | 31 | oral examination | - | - | - | - | + | - | - | SA, SD_Hue | - |
| Imfeld et al. 1988 | Regional | 155 | oral examination | DMFS | CPITN | - | - |  |  |  | - | - |
| Kamdem et al. 2017 | Regional | 992 | self-reported | - | - | - | - | + | + | - | questionnaire | - |
| Karsegard et al. 2001 | Regional | 35 | oral examination | - | PPD | + | PI |  |  |  | - | - |
| Menghini et al. 2002 | Regional | 25/47 | oral examination | DMFT | AL, PD | + | - | + | + | - | - | - |
| Mojon et al. 1998 | Regional | 120 | oral examination | Caries prevalence | CPITN | + | PI | + | + | - | - | - |
| Roccuzzo et al. 2023** | Regional | 275 | oral examination | - | PSI | - | - |  |  |  | SA, SD Hue | GOHAI |
| Roccuzzo et al. 2025* | Regional | 85 | oral examination | ICDAS, DMFT | PSI | - | mPBI, API |  |  |  | SA, SD_Hue | - |
| Schmidt et al. 2020 | National | 1033 | oral examination | - | PPD | + | - | + | + | + | - | - |
| Schneider et al. 2017 | National | NA | self-reported | - | - | + | - | + | + | + | - | - |
| Tennert et al. 2024** | Regional | 66 | oral examination | DMFT | PSI | - | API | + | + | + | SA, SD_Hue |  |
| Zitzmann et al. 2008 | National | NA | self-reported | - | - | + | - | + | + | - | - | - |
| Zitzmann et al. 2001 | National | NA | Self-reported |  | - | + | - | + | + | - | - | - |
| **Hospitals** | | | | | | | | | | | | |
| Campana R. and Verner Norgaard N. Diss. 2004 | Regional | 141 | oral examination | DMFT | BPE | + | - | + | - | - | - | - |
| Katsoulis et al. 2009 | Regional | 139 | oral examination | Caries prevalence | - | + | PI, GI | + | + | - | - | - |
| Meyer M. Diss. 1976 | Regional | 100 | oral examination | DMFT | - | + | - | + | - | - | - | - |
| Ohta et al. 2022 | Regional | 60 | oral examination | - | - | + | Number of microorganisms on the tongue dorsum | - | - | + | Glucose concentration | - |
| Rentsch A. Diss. 1993 | Regional | 216 | oral examination | Caries prevalence | - | + | - | + | + | - | - | - |
| Roehrich N. Diss. 1994 | Regional | 216 | oral examination | DMFT | CPITN | + | PI | + | + | - | - | - |
| Srinivasan et al. 2019 | Regional | 100 | oral examination | DMFT | CPITN | - | PI | + | - | - | - | - |
| Stuck et al. 1989 | Regional | 219 | oral examination | DF | Russell PI | + |  | + | - | - | - | - |
| **Long-term care facilities/Nursing homes** | | | | | | | | | | | | |
| Allenspach et al. 2024 | Regional | 389 | dental records | DMFT | - | + |  | + | - | - | - | - |
| Ammann R. Diss. 1988 | Regional | 150 | oral examination | DMFT | PBI | + | OHI | + | - | - | - | - |
| Anliker et al. 2023 | Regional | 180 | dental records | - | - | + | - | + | - | + | - | - |
| Bellomo et al. 2005 | Regional | 61 | oral examination | - | - | + | PI | + | - | - | - | - |
| Borg Bartolo et al 2021 | Regional | 47 | oral examination | DMFT | at least one tooth with >4mm | + | Oral Health Status (WHO 2013) |  |  |  | - | - |
| BrändliHolzer B. Diss. 2012 | Regional | 177 | oral examination | DMFT | - | + | OHI | + | - | - | - | - |
| Budtz-Jorgensen et al. 1996 | Regional | 235 | oral examination | Caries prevalence | - | + | - | + | - | - | - | - |
| Budtz-Jorgensen et al. 2000 | Regional | 233 | oral examination | Caries prevalence | - | - | PI | + | - | - | - | - |
| El Gedaily et al. 2024 | Regional | 503 | dental records | DMFT | PSR | + | + | + | - | - | - | - |
| GlauserPopaj L. Diss. 2009 | Regional | 276 | oral examination | DMFT | CPI, AL | + | OHI | - | - | - | - | - |
| Laas M. Diss. 2010 | Regional | 433 | oral examination | DMFT | - | + | - |  |  |  | - | - |
| Miodragovic M. Diss. 1980 | Regional | 216 | oral examination | - | Muhlemann and Mazor modified PM-Index | - | PI |  |  |  | - | - |
| Mojon et al. 1998 | Regional | 116 | oral examination | Caries prevalence | - | - | PI | + | - | - | - | - |
| Pazos E. Diss. 1999 | Regional | 290 | oral examination | - | CPITN | - | PI |  |  |  | - | - |
| Suter J. Diss. 1999 | Regional | 266 | oral examination | DMFT | CPITN | - | - |  |  |  | - | - |
| Triebold P. Diss. 1999 | Regional | 178 | oral examination | Caries prevalence | - | + | PI | + | - | - | - | - |
| Wirz et al. 1989 | Regional | 153 | oral examination | Caries prevalence |  | + | PI | + | + | - | - | - |

*reporting on the same study population

**reporting on the same study population

# Section 4. Quality Assessment

| Study | Were the criteria for inclusion in the sample clearly defined? | Were the study subjects and the setting described in detail? | Was the exposure measured in a valid and reliable way? | Were objective, standard criteria used for measurement of the condition? | Were confounding factors identified? | Were strategies to deal with confounding factors stated? | Were the outcomes measured in a valid and reliable way? | Was appropriate statistical analysis used? |
| --- | --- | --- | --- | --- | --- | --- | --- | --- |
| Allenspach et al. | y | y | y | Y | U | N | Y | Y |
| Ammann (Diss.) 1988 | Y | Y | Y | Y | NA | NA | Y | NA |
| Angst et al. 2024 | Y | Y | Y | Y | N | N | Y | Y |
| Anliker et al. 2023 | Y | Y | Y | Y | N | N | Y | Y |
| Bellomo et al. 2005 | Y | Y | Y | Y | N | N | Y | Y |
| Borg-Bartolo et al. 2021 | Y | Y | Y | Y | NA | NA | Y | Y |
| Borg-Bartolo et al. 2025 | Y | Y | Y | Y | Y | Y | Y | Y |
| Borg-Bartolo et al. 2025 | Y | Y | Y | Y | Y | Y | Y | Y |
| Brändli-Holzer (Diss.) 2012 | Y | Y | Y | Y | NA | NA | Y | NA |
| Budtz-Jørgensen et al. 1996 | Y | Y | Y | Y | N | N | Y | Y |
| Budtz-Jørgensen et al. 2000 | Y | Y | Y | Y | N | N | Y | Y |
| Campana and Verner Norgaard (Diss.) 2004 | Y | Y | Y | Y | NA | NA | Y | NA |
| Eggimann et al. 2024 | Y | Y | Y | Y | Y | Y | Y | Y |
| El Gedaily et al. 2024 | Y | Y | Y | Y | U | N | Y | Y |
| Glauser (Diss.) 2009 | Y | Y | Y | Y | NA | NA | Y | NA |
| Imfeld et al. 1988 | Y | Y | Y | Y | NA | NA | Y | Y |
| Kamdem et al. 2017 | Y | Y | Y | N | Y | Y | N | Y |
| Karsegard et al. 2001 | Y | Y | Y | Y | NA | NA | Y | Y |
| Katsoulis et al. 2009 | Y | Y | Y | Y | NA | NA | Y | Y |
| Laas (Diss.) 2009 | Y | Y | Y | Y | NA | NA | Y | NA |
| Menghini et al. 2002 | Y | Y | Y | Y | Y | Y | Y | Y |
| Meyer (Diss.) 1976 | Y | Y | Y | Y | NA | NA | Y | NA |
| Miodragovic (Diss.) 1980 | Y | Y | Y | Y | NA | NA | Y | NA |
| Mojon et al. 1995 | Y | Y | Y | Y | Y | Y | Y | Y |
| Mojon et al. 1998 | Y | Y | Y | Y | N | N | Y | Y |
| Ohta et al. 2021 | Y | Y | Y | Y | N | N | Y | Y |
| Pazos (Diss.) 1999 | Y | Y | Y | Y | N | N | Y | NA |
| Rentsch (Diss.) 1993 | Y | Y | Y | Y | NA | NA | Y | NA |
| Roccuzzo et al. 2023 | Y | Y | Y | Y | U | Y | Y | Y |
| Roccuzzo et al. 2025 | Y | Y | Y | Y | U | Y | Y | Y |
| Roehrich (Diss.) 1994 | Y | Y | Y | Y | NA | NA | Y | NA |
| Schmidt et al. 2020 | Y | Y | Y | Y | Y | Y | N | Y |
| Schneider et al. 2017 | Y | Y | Y | Y | Y | Y | Y | Y |
| Srinivasan et el. 2019 | Y | Y | Y | Y | N | N | Y | Y |
| Stuck et al. 1989 | Y | Y | Y | Y | Y | Y | Y | Y |
| Suter (Diss.) 1999 | Y | Y | Y | Y | NA | NA | Y | NA |
| Tennert et al. 2024 | Y | Y | Y | Y | Y | Y | Y | Y |
| Triebold (Diss.) 1999 | Y | Y | Y | Y | NA | NA | Y | NA |
| Wirz et al. 1989 | Y | Y | Y | Y | NA | NA | Y | NA |
| Zitzmann et al. 2001 | Y | Y | Y | Y | Y | Y | Y | Y |
| Zitzmann et al. 2008 | Y | Y | Y | Y | Y | Y | Y | Y |
| * Yes (Y), No (N), Unclear (U), Not applicable (NA) | | |  |  |  |  |  |  |

# Section 5. Data synthesis

**5.1 Edentulism**

Prevalence of Edentulism vs a) decade, b) setting

**Heterogeneity**

**Year**

**Setting**

**Level of data collection**

**Risk of bias**

**Leave one out analysis**

**5.2 Dental Status**

5.2.1 Dental caries

**Test for statistical significance when meta-analysis is performed using only 28-tooth-basis and including transformed data for 32-tooth-basis**

**Mean number of decayed teeth vs a) decade, b) setting (28-teeth-basis)**

**Mean number of decayed teeth vs a) decade, b) setting (32-tooth-basis)**

**5.2.2 Missing teeth**

**Test for statistical significance when meta-analysis is performed using only 28-tooth-basis and including transformed data for 32-tooth-basis.**

**Mean number of missing teeth vs a) decade, b) setting (28-tooth-basis)**

**Mean number of missing teeth vs a) decade, b) setting (32-tooth-basis)**

**Heterogeneity**

**Year**

**Setting**

**Risk of bias**

**Leave-one-out analyis**

**5.2.3. Filled teeth**

**Test for statistical significance when meta-analysis performed using only 28-tooth-basis and including transformed data for 32-tooth-basis**

**Mean number of filled teeth vs a) decade, b) setting (28-tooth-basis)**

**Mean number of filled teeth vs a) decade, b) setting (32-tooth-basis)**

**5.2.4 DMFT**

**Test for statistical significance when meta-analysis performed using only 28-tooth-basis and including transformed data for 32-tooth-basis**

**Mean DMFT vs a) decade, b) setting (28-tooth-basis)**

**Mean DMFT vs a) decade, b) setting (32-tooth-basis)**

**Heterogeneity**

**Year**

**Setting**

**Risk of BIas**

**5.3. Periodontal disease**

**Heterogeneity**

**Year**

**Risk of bias**

**5.4.1. Removable dental prosthesis**

**Prevalence of RDP vs a) decade, b) setting**

**Heterogeneity**

**Year**

**Setting**

**Risk of bias**

**5.4.2. Fixed dental prosthesis**

**Prevalence of FDP vs a) decade, b) setting**

**Heterogeneity**

**Year**

**Setting**

**Method of data collection**

**Risk of bias**
